# Pharmacological and Biological Study of Microencapsulated Probucol-Secondary Bile Acid in a Diseased Mouse Model

**DOI:** 10.3390/pharmaceutics13081223

**Published:** 2021-08-08

**Authors:** Susbin Raj Wagle, Bozica Kovacevic, Corina Mihaela Ionescu, Daniel Walker, Melissa Jones, Louise Carey, Ryusuke Takechi, Momir Mikov, Armin Mooranian, Hani Al-Salami

**Affiliations:** 1 The Biotechnology and Drug Development Research Laboratory, Curtin Medical School, Curtin Health Innovation Research Institute, Curtin University, Bentley, Perth, WA 6102, Australia; susbinraj.wagle@postgrad.curtin.edu.au (S.R.W.); bozica.kovacevic@postgrad.curtin.edu.au (B.K.); c.ionescu@postgrad.curtin.edu.au (C.M.I.); danieljcswalker@gmail.com (D.W.); melissa.a.jones@postgrad.curtin.edu.au (M.J.); louise.carey@student.curtin.edu.au (L.C.); 2 Hearing Therapeutics, Ear Science Institute Australia, Queen Elizabeth II Medical Centre, Nedlands, Perth, WA 6009, Australia; 3 School of Population Health, Curtin Health Innovation Research Institute, Curtin University, Bentley, Perth, WA 6102, Australia; R.Takechi@curtin.edu.au; 4 Department of Pharmacology, Toxicology and Clinical Pharmacology, Faculty of Medicine, University of Novi Sad, Hajduk Veljkova 3, 21101 Novi Sad, Serbia; momir.mikov@mf.uns.ac.rs

**Keywords:** diabetes mellitus, probucol, lithocholic acid, microcapsules, inflammation, oxidation

## Abstract

Probucol (PB) is a highly lipophilic drug with potential protective effects on pancreatic β-cells from inflammation and oxidation. PB has poor bioavailability and solubility, and despite many attempts, significant improvement in antidiabetic effects or absorption has yet to be discovered. Recently, the role of bile acids has been established in significant drug formulation stabilisation effects and as cell-penetrating agents. Promising results in pharmaceutical formulation studies on drug stability and release patterns when lithocholic acid (LCA) is conjugated with PB and sodium alginate (SA) have been demonstrated. Thus, this study aimed to develop and characterise PB microcapsules incorporating LCA and examine the biological effects of the microcapsules in vitro and in vivo. PB/LCA microcapsules were prepared using an encapsulation method, ionic gelation vibrational jet flow technology. LCA incorporation in PB microcapsules showed positive effects on β-cells with improved insulin release, antioxidant activity, and PB intracellular uptake. Diabetic mice gavaged LCA-PB microcapsules showed a significant reduction in diabetes signs and symptoms, better survival rate, reduced blood glucose levels, and pro-inflammatory cytokines, with an increase PB level in blood and tissues suggesting a potential therapy for treating diabetes mellitus.

## 1. Introduction

Diabetes mellitus (DM) is a metabolic disorder characterised by high blood glucose and inflammation and is categorised as type 1 (T1D) or type 2 (T2D) [1]. It is one of the most significant health challenges of this decade and has an enormous burden on economic and health systems globally [2,3]. The rates of diabetes across the world and in increasingly younger cohorts are rapidly increasing, with an annual cost of greater than AUD 15 billion in Australia alone [4].

Prediabetes is indicated by higher than normal blood glucose levels occurring before the onset of diabetes, and if diagnosed and treated effectively, it is possible to decrease the fast-growing rate of diabetes and related complications [5]. Reports indicate that almost one in three pre-diabetic patients will develop T2D if no effective interventions, such as early diagnosis, diet, and lifestyle changes, are employed [6,7]. Historically, T2D has been mainly regarded as dysregulation of blood sugar levels with less focus on related metabolic disturbances, inflammation, and oxidative stress.

One of the most significant causes of prediabetes is inflammation in combination with oxidative stress, which affects multiple organs, including the pancreas, and eventually develops into T2D [8,9]. Pre-diabetic inflammation has been connected to deteriorating diabetic symptoms, such as lipid dysfunction, insulin resistance, and obesity [10]. Pancreatic β-cell inflammation has also been connected to prediabetes and diabetes development directly; particularly as healthy β-cells have no natural antioxidant and anti-inflammatory defence capacity [11]. Therefore, a way to prevent prediabetes from developing into diabetes is not only controlling high blood glucose levels but addressing underlying inflammation, oxidative stress, and pancreatic β-cell damage. This is posited to be possible using adjuncts with antioxidant and anti-inflammatory properties that ultimately protect pancreatic β-cells from inflammation and oxidation.

Probucol (PB) is a highly lipophilic drug with potent antioxidant and anti-inflammatory properties. It has potential applications in prediabetes treatment due to protective effects on pancreatic β cells from inflammation and oxidation [12,13]. Despite this significant potential, its pharmacokinetics and tolerability in current dosage forms pose substantial challenges. PB has a variable absorption profile, poor water-solubility, low oral bioavailability, low clinical efficacy, and severe adverse effects [14,15,16]. One potential approach to overcome these challenges is by optimising a formulation that protects PB in the gastrointestinal tract, allowing for precise release at the absorption site and enhanced intestinal permeation. All of this can be achieved by incorporating PB with bile acids, known permeation-enhancing agents [17], using artificial cell microencapsulation (ACM) technology. ACM is a technique mainly used to improve the oral delivery of lipophilic drugs by encapsulating them with biodegradable polymers [18,19].

Bile acids primary function is to support solubilisation and digestion of lipids. Recently they have been shown to improve oral drug delivery as absorption and permeation-enhancing agents as well as improving drug stability [20,21,22]. The secondary bile acid lithocholic acid (LCA) has shown anti-inflammatory and formulation-stabilising effects in atherosclerosis [23]. Our group studied the impact of PB-LCA microcapsules on pancreatic β-cells and found positive effects on cell viability, including anti-inflammatory effects and increased bioenergetic parameters [24,25].

Thus, this study aimed to form microcapsules integrating PB and LCA using sodium alginate (SA) as a polymer and examine the potential of these developed microcapsules using in vitro and in vivo tests, appropriate for oral delivery in T2D.

## 2. Materials and Methods

### 2.1. Materials

PB (99.89%), sodium alginate low-viscosity (99%), LCA (≥95%), and alloxan (>98%) were obtained from Sigma-Aldrich CO. (St. Louis, MO, USA). Calcium chloride dihydrates (CaCl_2_·2H_2_O, 98%) were purchased from Scharlab S.L (Barcelona, Spain). Ultrasonic gel was purchased from the Australian Medical Association (Perth, WA, Australia). 20,70-Dichlorofluorescin diacetate (DCFH-DA) was purchased from Sigma-Aldrich Corporation CO., (St. Louis, MO, USA) and 2,2′-Azobis-2-methyl-propanimidamide, dihydrochloride (AAPH) purchased from Sapphire Bioscience (Redfern, NSW, Australia).

### 2.2. Drug Preparations

Stock suspensions of sodium alginate (1%), PB (1%), and LCA (3%) were prepared by solubilising the PB and LCA crystal powder with 2.5% ultrasonic water-soluble gel, thus producing stock mixtures for the formulations. The 6% *w/v* CaCl_2_ standard solution was prepared by dissolving CaCl_2_ in deionised water. Alloxan was mixed with sterile saline before injecting it into mice.

### 2.3. Microencapsulation Preparation, Formulation, and In Vitro Study

F1 as formulation-1 (PB-SA) and F2 as formulation-2 (PB-LCA-SA) microcapsules were prepared from the stock solutions as per our well-established laboratory method using the Ionic Gelation Vibrational Jet Flow Technology on a Büchi encapsulator (Büchi Labortechnik, Flawil, Switzerland) [26,27,28,29,30,31,32]. Briefly, different parameters were set in a frequency range of 1000–2000 Hz with a flow rate of 6 mL/min with a constant air pressure of 300 mbar. From the stock solution, formulations with and without LCA were made up to a final concentration in a PB: SA: LCA ratio of 1:30:3, respectively. Microcapsules were collected in a CaCl_2_ ionic gelation bath. Finally, microcapsules were dried using a stability chamber (Angelantoni Environmental and Climatic Test Chamber, Massa Martana, Italy).

Microcapsule morphology and surface topography were analysed via optical microscopy (OM) (Nikon SM2800, Tokyo, Japan), scanning electron microscopy (SEM) (Neon 40EsB FIB-SEM; Zeiss, Oberkochen, Germany), energy dispersive X-ray spectra (INCA X-Act; Oxford Instruments, Abingdon, U.K.) and micro-CT (SkyScan 1172A Micro-CT, Kontich, Belgium) [33,34]. Briefly, for OM, 10 freshly prepared microcapsules from each group were dried using a stability chamber, randomly selected, and put onto a glass slide mounted to a calibrated scale. Likewise, for SEM and EDXR, the dried capsules were mounted on an aluminium stub and coated with a layer of 3 nm platinum under vacuum.

Rheological parameters were analysed using a Visco-88 viscometer (Malvern Instruments, Malvern, UK) using freshly made stock mixtures. Aliquots of each formulation were placed in the instrument cap at room temperature, and viscosity, shear stress, and rate were measured at different rotation speeds.

Thermal and chemical analyses were undertaken by differential scanning calorimetry (DSC) (DSC 8000, PerkinElmer Inc., Waltham, MA, USA) and Fourier transform infrared spectroscopy (FT-IR) (Waltham, MA, USA). Briefly, 5 mg of dry microcapsules were put in a sealed aluminium pan and heated at 30 °C per minute at a flow rate of 20 mL/minute under a nitrogen atmosphere in the 30–250 range. For FT-IR, the spectra of microcapsules were measured in the transmission frequency range of 450–4000 cm^−1^.

Zeta potential was analysed using a 3000HS Zetasizer (Malvern, UK), and size distribution in the dispersion system was collected by using photon correlation spectroscopy (Fraunhofer scattering technique, Malvern, UK) [29,35]. Zeta potential measurements were carried out on our formulations prior to encapsulation using our established methods [29].

Mechanical resistance was measured as per established methods. Briefly, 25 dry microcapsules were placed in 20 mL pH 7.8 phosphate buffer on a shaker and stirred at a frequency of 150 rpm for 24 h (Boeco Company, Hamburg, Germany). After 24 h, the number of damaged microcapsules was counted and compared to the initial number of capsules [28,29,36].

Microcapsules swelling properties were examined by placing 200 mg of dried microcapsules in 20 mL phosphate buffer at two different pH values (3 and 7.8) at room temperature for 6 h. The selection of temperature and pH values was based on our previous work [34,35]. The net wet weight of swollen microcapsules was calculated by weighing on a dynamic balance immediately after blotting them with filter paper (Whatman #40) to remove excess moisture. The microcapsules swelling index was determined in percentages as described [32,37].

Microcapsules’ buoyancy was determined by placing 100 microcapsules in 200 mL of simulated intestinal fluids that consisted of enzyme-based phosphate buffer (pH 7.8). The solution was stirred for 6 h at 50 rpm at a set temperature of 37.5 °C on a USP dissolution apparatus (24, type II). The number of floating microcapsules was examined visually and counted at the end [25,38].

NIT-1 pancreatic β-cells were used to measure insulin secretion, antioxidant efficiency, and PB cell permeability. The cells were grown in Dulbecco’s Modified Eagle’s Media (DMEM-Sigma Andrich, St Louis, MO, USA) with 10% fetal bovine serum (FBS-Thermo Fisher, Melbourne, Victoria, Australia) as per our standard protocol [24,25,39]. Briefly, to test the effect of F1 and F2 microcapsules, 1.5 × 10^6^ cells/mL NIT-1 cells were cultured at 5.5 mM (normoglycemic state) and 35 mM glucose (hyperglycaemic state) in DMEM and treated with PB- loaded microcapsules for 48 h. After 48 h, microcapsules were removed, and aliquots of media were tested as described [24] for glucose levels, antioxidant levels, and PB permeability.

The level of glucose-stimulated insulin released was measured using a Mercodia Insulin ELISA kit (USA) and BD FACSCanto II (BD Biosciences, Franklin Lakes, NJ, USA) as per our established method using the Attune Acoustic Focusing Flow Cytometer [40]. For the antioxidant assay, cells were incubated with PB-loaded microcapsules in media with glucose concentrations of 5 and 35 mM for 48 h. After 48 h, cells were treated with DCFH-DA (4.6 mM) (dichloro-dihydro-fluorescein diacetate) and AAPH (18.44 Mm) (2,2-azobis(2-amidinopropane) dihydrochloride) as described [41]. The fluorescence was measured using an Enspire Multimode Plate Reader (PerkinElmer, Hopkinton, MA, USA) at 485 nm absorption and a 538 nm emission wavelength.

For PB cellular uptake, cells were plated in double-chamber tissue culture pates (Transwell, 24-well filter chambers, 0.4 μm pore size membrane; Costar, Coring Inc., New York, NY, USA) with PB-loaded microcapsules placed on the bottom of the insert for 48 h at a glucose concentration of 35 mM. After 48 h, microcapsules were removed, and the media was collected for the analysis from the upper compartment, porous membrane, and lower compartment of the transwell for high-pressure liquid chromatography (HPLC) analysis [42]. PB was analysed by HPLC system (Pump model: LC-20AT, UV detector: SPD-20A and injector model: SIL-20AC, Shimandzu Corporation, Kyoto, Japan) at a wavelength of 242 nm as described, and data were standardised as ng of PB per 1.0 × 10^5^ cells [43].

### 2.4. In Vivo Studies

According to the Australian Code of Practice for the care and use of animals for scientific purposes, male mice (6 weeks BALB/c) were brought from the Animal Resources Centre (Perth, WA, Australia) and tested in an animal facility at Curtin University, Perth, WA. The experiment is approved by the Animal Ethics Committee, Curtin University (ARE2017-7). All experiments were performed according to the Australian Code of Practice for the care and use of animals for scientific purposes.

All mice were fed with a high-fat diet (HFD) consisting of feed enriched with 30% (*w/w*) lard, 15% (*w/w*) fructose, and 0.5% (*w/w*) cholesterol (Specialty Feeds, Perth, Australia). for twelve weeks. At week 4, 50 mg/kg/bodyweight of alloxan was given subcutaneously to accelerate the development of a pre-diabetic condition [44,45,46,47]. After a week of alloxan injections, all mice showed pre-diabetic signs; mice demonstrating a blood glucose level of ≥11 mmol/L, together with signs and symptoms of diabetes, were considered pre-diabetic [44,48]. Following this, twenty-four mice were arbitrarily divided into four equal groups. Group 1 was orally gavaged with empty microcapsules, group 2 with free PB (powder) (PB: 80 mg/kg/day), group 3 with PB microcapsules (PB: 80 mg/kg/day), and group 4 with PB-LCA microcapsules (PB: 80 mg/kg/day and LCA: 40 mg/kg/day). The dosage of microcapsules was calculated according to our previous studies [27,49].

**Figure 1:**
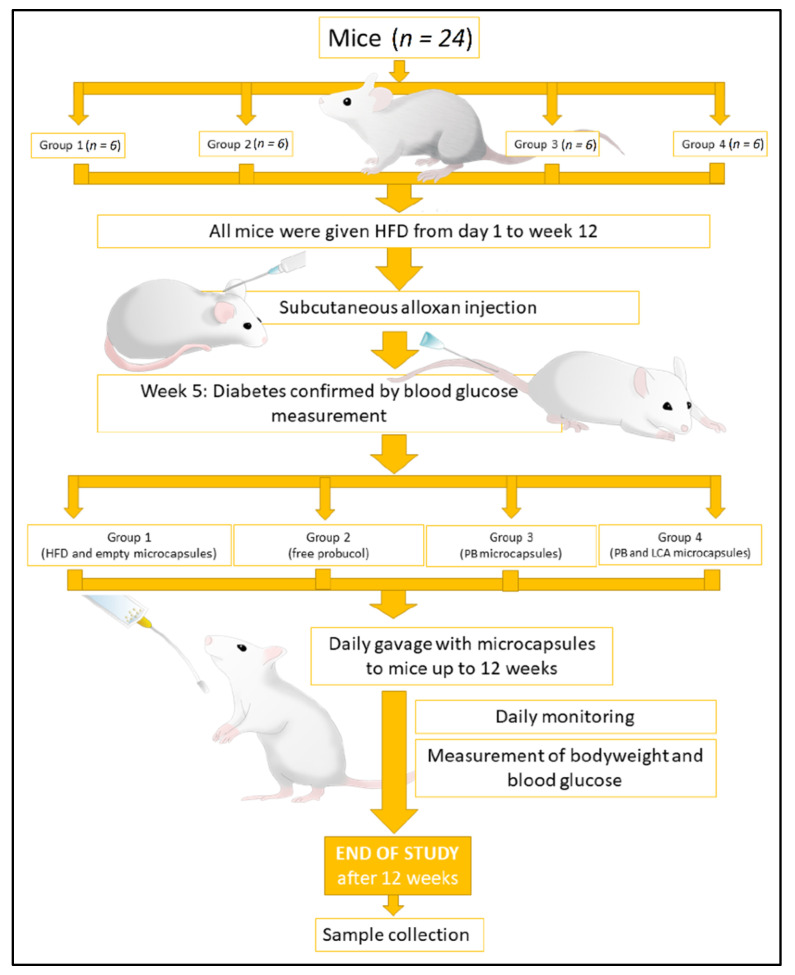
Summary of in vivo study.

All mice were kept in a 12 h light and dark cycle room (22 °C) with *ad libitum* access to water and HFD. At the end of the 12-week experiment, all mice were anaesthetised with isoflurane and euthanised by cervical dislocation as per approved animal protocols. Blood, tissue, and faeces were collected and used for PB and blood glucose analysis. Overall, the in vivo study conducted on mice from week 0 to week 12 was divided into four different groups, six in each group, and is summarised in Figure 1.

### 2.5. PB HPLC (High-Performance Liquid Chromatography) Analysis

PB standard concentrations from 0.04 to 0.8 mg/mL were made in acetonitrile: water (98:2% *v/v*) ratio. The flow rate was kept at 1.5 mL/min with an auto-sampler injection volume of 40 µL. The HPLC setup was a low-pressure gradient Shimandzu Prominence HPLC system (Pump model: LC-20AT, UV detector: SPD-20A and injector model: SIL-20AC, Shimandzu Corporation, Kyoto, Japan) at a wavelength of 242 nm. The separation was performed using a Phenomenex C-18 reverse phase column with an internal diameter of 4.5 mm and a length of 25 cm attached with the guard column.

PB extraction from blood and other tissues was performed by mixing serum or tissues with ice-cold acetonitrile in a 1:1 ratio. After centrifugation, 40 µL of purified serum (supernatant) was mixed with 160 µL mobile phase acetonitrile, gently vortexed for 20 s, and then centrifuged at 15,000 rpm for 20 min. After this, 20 µL of supernatant was transferred for analysis to an auto-sampler HPLC vial.

### 2.6. Plasma Blood Glucose, Survival Rate, and Cytokines Analysis

Blood glucose levels were measured for all mice using an Accu-check Performa glucometer from vein tail puncture (Roche Laboratories, Gartenstrasse, Basel, Switzerland), based on our well-established methods [50]. Mouse survival was supervised daily. Plasma cytokines were measured as per our established method using a cytokine bead array kit (BD Biosciences, USA) using Attune Acoustic Focusing Flow Cytometer (Life Technologies, Carlsbad, CA, USA) [39,49,51].

### 2.7. Statistical Analysis

All the results are presented as mean ± standard deviation (SD). Statistical data were evaluated using ANOVA or parametric/non-parametric analysis, as appropriate using GraphPad Prism Version X8.2 (GraphPad, San Diego, CA 92108, USA). The level of significance was considered at *p* < 0.01 or *p* < 0.05.

## 3. Results

### 3.1. Formulation Analysis: Microcapsules Morphology, Size, Surface, Internal Complexity, and Thermal Characterisation

**Figure 2:**
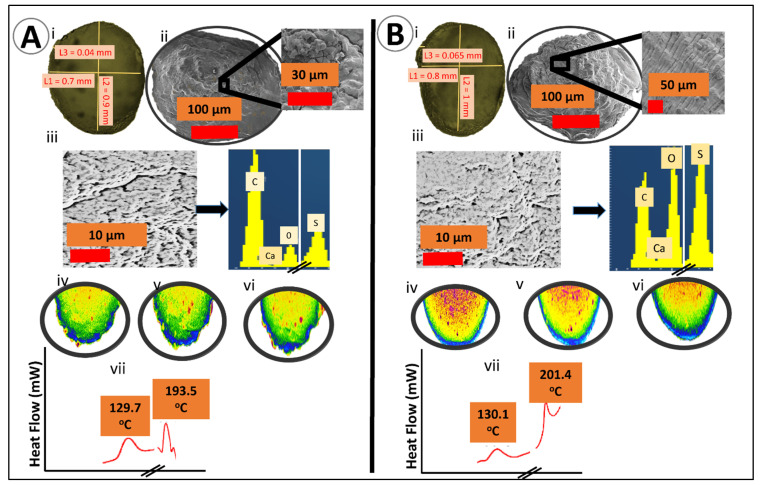
(**A**) F-1; PB-SA microcapsules; (i) optical image, (ii) SEM image at 100 μm, (iii) EDXR spectra and corresponding analysis, (iv–vi) micro-CT-analysis reflecting the internal structure and morphology, (vii) DSC thermograms. (**B**) F2; PB-LCA-SA microcapsules; (i) optical image, (ii) SEM image at 100 μm, (iii) EDXR spectra and corresponding analysis, (iv–vi) micro-CT-analysis reflecting the internal structure and morphology, (vii) DSC thermograms.

Figure 2A,B show the images of OM, SEM, EDXR, and Micro-CT of F1 and F2 microcapsules, respectively. In Figure 2(Ai,Bi), the horizontal diameter (L1), vertical diameter (L2), and membrane width (L3) were measured. Consistency between F1 and F2 microcapsules in both shape and size was found. The optical microscopy images, Figure 2(Ai,Bi), displayed spherical, opaque, and discrete microcapsules with homogenous particle size distribution throughout the microcapsules, which was further supported by SEM studies (Figure 2(Aii,Bii)). The surface crystal deposits and composition of the microcapsules were analysed using EDXR, and the spectra showed the presence of different atoms of sulfur, calcium, oxygen, and carbon. These deposits are present on the surface of both microcapsules Figure 2(Aiii,Biii), with the presence of sulfur specific to PB, demonstrating the presence of PB on the surface of the microcapsules. Figure 2A,(Biv–vi) display the micro-CT analysis of F1 and F2 microcapsules, respectively. F2 microcapsules presented as denser (inner core) with a distinct outer surface. Likewise, Figure 2(Avii,Bvii) show DSC spectra of F1 and F2 microcapsules, respectively.

### 3.2. Chemical Stability, Zeta Potential, and Mean Particle Size Analysis

Table 1 shows the zeta potential and particle size distribution of F1 and F2 formulation prior to encapsulation. The presented data suggest no significant effect on the particle size distribution and zeta potential by the addition of LCA.

The FT-IR spectra peaks are presented in Table 1. FT-IR showed characteristic peaks of F1 microcapsules at 3298, 1416, 1309, and 1024 cm^−1^ and F2 microcapsules at 3340, 1418, 1309.3 and 1024 cm^−1^, suggesting the chemical compatibility was maintained post-microencapsulation.

### 3.3. Rheological Studies

**Figure 3:**
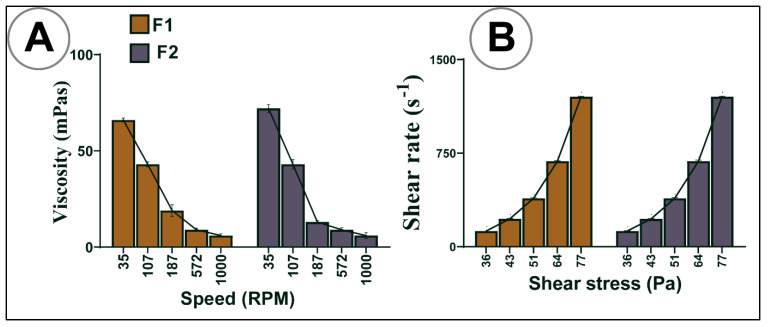
Rheological parameters (**A**) influence of speed on viscosity, (**B**) influence of shear stress on shear rate. *N* = 3, mean ± SD.

Table 2 and Figure 3A,B show the rheological behaviours of both formulations under various mixing speeds. The addition of LCA to F1 did not considerably alter the rheological parameters (viscosity, torque, shear stress, and shear rate). The pattern showed that as speed increased, viscosity decreased (Figure 3A), and torque increased (Table 1). Figure 3B indicates that shear stress and shear rate had a direct relationship; when one increased, the other also increased, suggesting non-Newtonian thixotropic behaviour [52].

### 3.4. Buoyancy, Mechanical Resistance, and Swelling Studies

**Figure 4:**
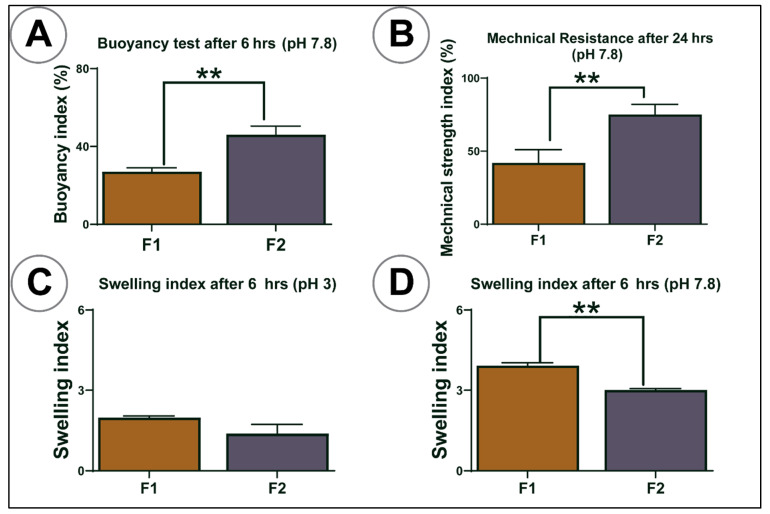
**(A**) Buoyancy index at pH 7.8, (**B**) mechanical resistance at pH 7.8, (**C**,**D**) swelling index at pH 3 and (**D**). F1 = PB-SA microcapsules, F2 = PB-LCA-SA microcapsules. *N* = 3, mean ± SD. *p* < 0.01 **.

Figure 4A–D shows the buoyancy, swelling studies at 6 h, and mechanical resistance index of both control and test microcapsules at 24 h. For buoyancy, the percentage of floating microcapsules was calculated over 6 h. In mechanical resistance testing, the percentage of the microcapsules’ ability to resist mechanical stress was measured over 24 h. The results showed that the addition of LCA enhanced buoyancy (*p* < 0.01) (Figure 4A) and decreased swelling (25 °C at pH 7.8) (*p* < 0.01) (Figure 4D) but was not affected at pH 3 (Figure 4C), where better mechanical strength was observed (*p* < 0.01) (Figure 4B). LCA addition to F1 microcapsules made capsules more stable and improved the microcapsule capacity to interact with the gut contents and maintain pH-targeted delivery (pH > 7), which is in line with our previous studies [30,31,33].

### 3.5. In Vitro Study: Pancreatic β Cells Insulin, Antioxidant Assay, and PB Cellular Uptake

**Figure 5:**
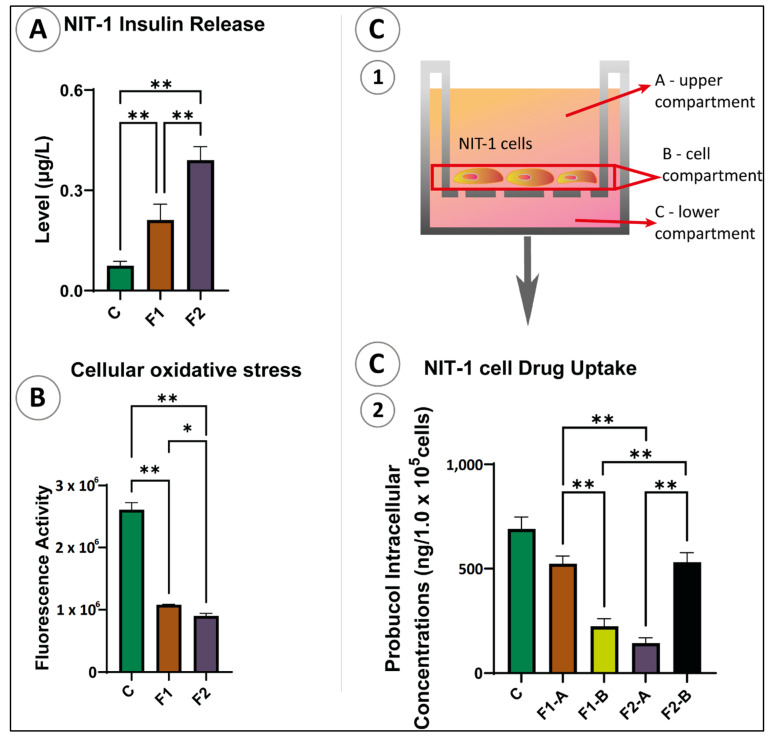
(**A**) Insulin release, (**B**) level of oxidative stress, (**C1**) transwell, (**C2**) PB cellular uptake of NIT-1 cell after exposure to the microcapsules; F1 = PB-SA microcapsules, F2 = PB-LCA-SA microcapsules. *N* = 3, mean ± SD. *p* < 0.05 *, *p* < 0.01 **.

Figure 5 shows the total insulin production (Figure 5A), antioxidant assay (Figure 5B), and PB cellular uptake (Figure 5C) of pancreatic β-cells at 35 mM glucose concentration over 48 h for untreated cells (control—empty microcapsules), F1, and F2 microcapsules. Figure 5A shows F1 microcapsules significantly increased insulin level (*p* < 0.01). Likewise, in Figure 5B, treated NIT-1 cells showed lower fluorescence activity, which indicates more significant antioxidant activity. The LCA addition on F1 microcapsules not only enhanced insulin secretion but also showed high antioxidant activity (*p* < 0.01).

In Figure 5(C2), F1 PB concentration was higher in the ‘A’ upper compartment, compared with the B compartment, whereas in F2, PB concentration was higher in compartment B compared with compartment A, which showed that LCA addition to F1 microcapsules significantly increased intracellular uptake of PB (compartment B). The PB concentration was below the limit of detection in the lower compartment C in both F1- and F2-treated cells.

### 3.6. In Vivo Studies

#### Bodyweight, Water Consumption, Survival Rate, Blood Glucose, and Cytokine Analysis

**Figure 6:**
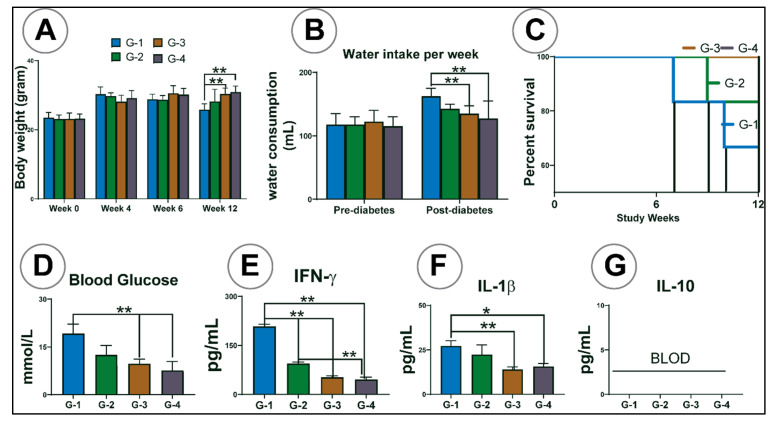
(**A**) Mice body weight at different weeks, (**B**) water consumption rate, (**C**) survival rate, (**D**) blood glucose measurement at the end of the experiment, (**E**) concentration of pro-inflammatory cytokines in plasma, interferon-γ, (**F**) concentration of pro-inflammatory cytokines, interleukin-1β, and (**G**) concentration of anti-inflammatory cytokines, interleukin-10. G-1 = group one as a diabetes control mice fed with the high-fat diet, G-2 = mice treated with free PB, G-3 = mice treated with PB-SA = F1 microcapsules, G = 4 mice treated with PB-LCA-SA = F2 microcapsules. BLOD = below the limit of detection. Data are presented as mean ± SD. *p* < 0.05 *, *p* < 0.01 **.

Figure 6A shows the average mice body weight at week 0, week 4, week 6, and week 12. At week 0, all the mice had similar body weights irrespective of the group (G). When fed with a high-fat diet, the bodyweight of all mice was increased. However, at the end of week 12, G-1 mice’s body weight decreased significantly compared to G-3 and G-4 (*p* < 0.01).

Figure 6B displays the water consumption rate before and after diabetes onset. The water consumption rate was similar between the groups before inducing diabetes. However, following the induction of diabetes, G-1 mice (untreated control) took in more water compared to other groups (*p* < 0.01).

Figure 6C shows the survival rate among all the groups, and the survival rate was high for G-3 and G-4, followed by G-2 and lowest for G-1.

Diabetes induction caused a significant increase in blood glucose levels. Compared to the control group (G1), blood glucose levels decreased in PB powder-treated G-2 and further decreased in PB microcapsule-treated G-3 (*p* < 0.01), with the PB/LCA microcapsule-treated G-4 showing the greatest reduction in blood glucose levels (*p* < 0.01) (Figure 6D). Similarly, groups treated with microcapsules and free PB showed lower serum levels of pro-inflammatory cytokines (IFN-γ and IL-1β) (Figure 6E,F) (*p* < 0.01), while the level of anti-inflammatory cytokines, IL-10 were below the limit of detection in all four groups (Figure 6G). The most significant decrease in IFN-γ was noticed in G-4 compared to other groups. Overall, the results also showed no significant difference in body weight, water intake, survival rate, blood glucose, and pro-inflammatory cytokines expression between mice gavaged with PB-loaded G-3 and PB-LCA-loaded G-4 microcapsules.

### 3.7. Drug Analysis in Different Organs and Tissues

**Figure 7:**
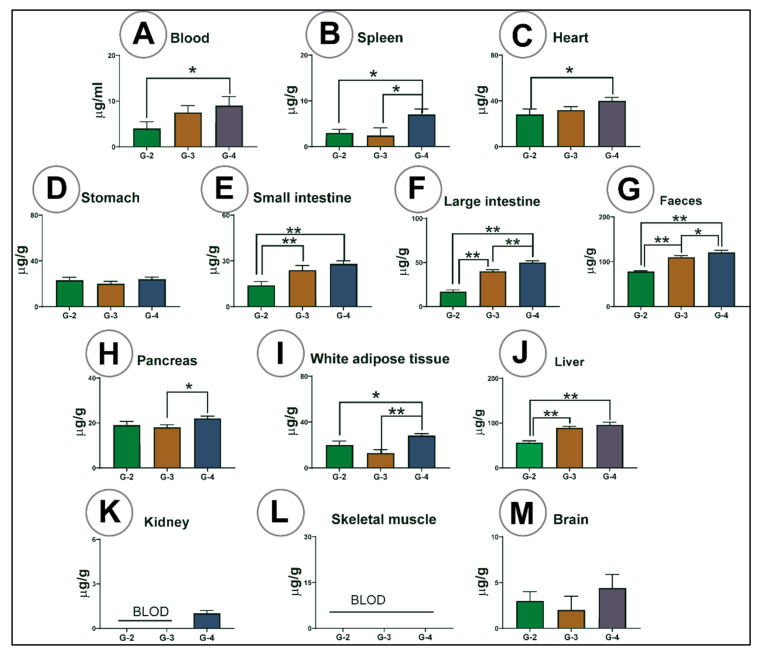
PB concentration (µg/mL or µg/g) in blood (**A**), spleen (**B**), heart (**C**), stomach (**D**), small intestine (**E**), large intestine (**F**), faeces (**G**), pancreas (**H**), white adipose tissue (**I**), liver (**J**), kidney (**K**), skeletal muscle (**L**) and brain (**M**). G-2 = mice treated with free PB, G-3 = mice treated with PB-SA = F1 microcapsules, G = 4 mice treated with PB-LCA-SA = F2 microcapsules. BLOD = below the limit of detection. Data are presented as mean ± SD. *p* < 0.05 *, *p* < 0.01 **.

Figure 7 shows PB concentration in different organs and tissue. G-4 and G-3 mice treated with PB microcapsules showed higher PB concentration in blood, liver, heart, brain, small intestine, large intestine, and faeces than G-2 receiving free probucol powder. The drug was detected in the kidneys only in G-4 at a low concentration. PB was not detected in skeletal muscle in any group. In comparing G-3 and G-4, PB levels were higher in the pancreas, spleen, large intestine, faeces, and white adipose tissues in G-4 mice, suggesting that bile acid acted as a permeation-enhancing agent and increased the oral uptake of drug [53]. Although not statistically significant, PB concentration was higher in the brain tissue treated with PB-LCA microcapsules. All groups showed similar PB levels in the stomach, indicating no drug was lost or degraded until it reached the intestines.

## 4. Discussion

Through OM, SEM, and surface elemental examination, results showed that the integration of LCA did not alter the shape, size, and surface elemental distribution of PB in microcapsules. These findings are consistent with our previous results demonstrating that our microencapsulation method is robust and uniform regardless of formulation and microencapsulation excipients [25,30,34]. Even though F1 and F2 microcapsules have similar morphology (shape, size, and elemental distribution), X-ray micro-CT scanning Figure 2(Aiv–vi,Biv–vi) displayed a significant difference in internal complexity between F1 and F2. F2 microcapsules showed denser internal complexity with a well-defined outer membrane, probably due to bile acid distribution inside the core of microcapsules rather than on the surface. This can act as a membrane-stabilising and structural support agent to increase the solubility and permeation of drugs [39]. These results support previous studies showing that bile acid incorporation did not change the shape and size but does alter the outer membrane and the internal complexity [27,38].

The rheological parameters exhibited indicated that both the formulations behaved as non-Newtonian fluids with thixotropic- pseudoplastic behaviour, and that the addition of LCA did not alter the rheological properties, which is in line with other studies [34,54,55]. This flow behaviour is essential to determine the pharmacodynamics and pharmacokinetics parameters in terms of pH-targeted delivery as well as transit time after an oral dose [56,57]. This behaviour is important in providing less mechanical stress on the encapsulator nozzles as formulation fluid is passing through, allowing for uniform capsule size formation and stability profiles.

DSC was used to determine the thermo-analytical behaviour of compounds. DSC spectra of F1 microcapsules showed two distinct peaks at 129.7 and 193.5 °C. Likewise, the DSC spectra of F2 microcapsules presented two different peaks at 130.1 and 201.4 °C, suggesting the thermal stability of the microcapsules’ constituents. The prepared microcapsules showed no significant change in thermal characteristics in PB formulations, even when LCA is added, suggesting that the incorporation of the bile acid LCA did not compromise or negatively impact molecular thermodynamic or PB thermostability within the capsules. The DSC findings suggest that the encapsulation technology and bile acid incorporation were robust and maintained the thermal integrity of the drug PB. Fourier transform infrared imaging was used to support DSC results, which determines the chemical composition of the compound. In line with the DSC results, Table 1 presents the assignation of the characteristic FT-IR peaks based on our published analytical FT-IR findings. This showed no significant chemical alternation at the molecular level occurred during the process of microencapsulation even after the addition of LCA in PB formulation, which is consistent with our previous studies on PB encapsulation [34,58]. The FT-IR spectra results corresponding at 1416 (C–H functional group) and 1309 cm^−1^ (S=O functional group) represent the characteristic peaks and specific chemical structure of PB in F1. The spectra peak read at 1418 and 1309.3 cm^−1^ in F2 formulation suggest that there was no thermal or chemical reaction between PB and the polymer matrix that could interfere with the drug and alter its physicochemical properties.

The analysis of surface charge is important to measure the electrokinetic stability of the particles in a colloidal suspension. Such stability is normally measured in terms of zeta potential; the higher the zeta potential value, the greater the particle stability because of high electrostatic repulsion between the particles [59]. Size measurement was used to determine the colloidal dispersion and uniformity of the formulation. This characteristic is important as it affects molecule interaction when encapsulating, thus affecting capsule shape and size. The incorporation of LCA did not significantly change zeta potential or particle size, which is consistent with our previous work and supports the kinetics of encapsulated drug release (Table 1) [26,60].

SA is a biodegradable polymer that protects the drug, and its physicochemical properties are impacted by gastrointestinal pH values [19,61]. The pH values examined were chosen based on previous studies conducted in our lab, which typically reflect the distal segment of the small intestine from where the absorption of the antidiabetic drug, either alone or together with bile acids, should occur [22,44,62]. The membrane-stabilising studies showed microcapsules had increased swelling properties in higher pH values for both formulations indicating that SA undergoes extensive swelling at high pH due to higher water uptake caused by increasing its porosity, water infiltration, and solubilisation of the polymer [63]. The addition of LCA significantly decreased the swelling features of microcapsules, mainly at pH 7.8, which supports controlled drug release in terms of in vivo applications. Likewise, the mechanical strength results also revealed that the addition of LCA significantly enhanced PB microcapsules’ strength, which suggests LCA prevents sudden microcapsule rapture and makes them more resistant to mechanical stress at pH 7.8. The buoyancy index showed high buoyancy for F2 microcapsules compared to F1 microcapsules (*p* < 0.01) under simulated intestinal fluid conditions, which means most F2 microcapsules float and can be retained in the ileum, supporting the gradual release of PB in the distal segment of the small intestine. Overall, swelling, mechanical index, and buoyancy results support our hypothesis that bile acids act as membrane-stabilising agents, especially when microencapsulated with SA. Better control and targeted PB release (at pH 7.8) can prevent premature drug loss due to rapid changes in GI pH values (1.5–7.8) in oral administration [35,60].

Throughout in vitro studies, PB-enhanced insulin production and antioxidant activity, with LCA-containing microcapsules producing significant results at the tested conditions. These results suggest that insulin secretion and antioxidant activity in response to PB treatment is formulation-dependent and enhanced by the presence of LCA, similar to our previous findings [43]. The improved β-cell function brought by PB microcapsules seems to be directly correlated with an increased IL-10 anti-inflammatory profile and decreased IFN-γ pro-inflammatory profile, and bioenergetics parameters [24,25]. Moreover, drug intracellular assays further support the improved β-cell activity treated with F2 microcapsules. Intracellular PB uptake was higher when LCA was incorporated, indicating LCA microcapsules have the capacity to enhance cell permeation through the cells’ phospholipid bilayer resulting in a higher intracellular PB level. In another study, PB uptake was similar among the treated groups, and the bile acid CDCA (chenodeoxycholic acid) incorporation did not cause any significant alternations of PB cell permeation [43]. The study suggested that the PB absorption level remains constant and independent of formulation excipients. Conversely, our study suggests that the pancreatic β-cell absorption of PB intracellularly is dependent on the formulation. This significant positive impact may be due to the strong formulation-stabilisation effects that support the controlled release of the drug [25]. These properties of LCA increase PB uptake by β-cells that subsequently enhance the PB permeation into the cells and improves other biological functions such as increasing insulin secretion and increasing antioxidant activity. However, more studies are needed in the future to investigate the influence of the intracellular mechanisms of PB-LCA on β-cells.

To the best of our knowledge, PB encapsulation with LCA effects on blood glucose, anti-inflammatory action, and PB absorption in vivo have not yet been explored. Generally, diabetic mice lost weight and increased water intake when glucose levels rose (Figure 5). In our study, oral gavage of PB microcapsules reduced signs and symptoms of diabetes and increased survival rate (Figure 4B,C and Figure 6A). All the groups treated with PB showed lowered blood glucose levels, but a significant reduction was observed only in the group treated with F2 microcapsules. The reason may be due to F2 microcapsules stimulating the uptake of glucose and reducing inflammatory cytokines, which is possibly due to the positive antidiabetic and anti-inflammatory effects of PB restoring the function, morphology and improving the survival of pancreatic β-cells [64]. However, histological examination of pancreatic β-cells is also needed to support the mentioned hypothesis, which is outside the scope of this study.

Plasma inflammatory biomarkers, such as interferon-gamma (IFN-γ), have been connected with diabetes development and progression with the use of pro-inflammatory cytokines suggested to measure disease progress [65]. In this study, oral gavage of PB microcapsules decreased pro-inflammatory cytokines, IFN-γ and IL-1β, whereas the expression of anti-inflammatory cytokine IL-10 was not detected (probably below the limit of detection). The results have shown that LCA incorporation in PB microcapsules decreased pro-inflammatory cytokines, but the decrease was not statistically significant. Mooranian et al. have demonstrated findings similar to our study that the ursodeoxycholic bile acid decreases pro-inflammatory cytokines, but this was not statistically significant, and likewise, anti-inflammatory cytokines were not detected [47,49]. This may be due to either the short time duration of the experiment or different cellular reactions to various excipients of the microcapsules [66]. In our previous in vivo study, when NIT-1 pancreatic β-cells were treated with PB-LCA microcapsules, a lower level of pro-inflammatory cytokines (IFN-γ and IL-1β) and high levels of anti-inflammatory cytokine (IL-10) were detected (paper under publication). Thus, PB alone or microencapsulated with LCA showed a potent anti-inflammatory effect, decreased the incidence of diabetes, and improved diabetic symptoms, blood glucose levels, and survival rate [64]. These results show promise in terms of application for PB in T2D therapy. However, since this is the first and preliminary study, more extended studies will be conducted in the near future.

Mice gavaged with PB-LCA microcapsules showed a high level of PB in most of the analysed tissue, including blood, liver, heart, brain, pancreas, spleen, small intestine, large intestine, faeces, and white adipose tissue, indicating that specially developed LCA-PB microcapsules have promising effects in diabetes prevention and post-diabetes complications. All three groups were given the same PB dose, so they showed equal concentration in the stomach. However, G-4 mice gavaged with PB-LCA microcapsules had higher PB levels in the small intestine and large intestine, indicating gastro-protection until the drug reaches the absorption site in the gut, which demonstrates that bile acids enhance absorption and improve stability of the drug [67]. Likewise, we found a higher level of PB in blood and other tissues, which proves that PB-LCA microcapsule enhanced drug absorption and increased bioavailability and tissue accumulation, probably through the inhibition of the efflux system leading to increased PB net absorption and cellular permeation [68,69]. PB was detected in very small concentrations in the kidney in G-4 mice and not in other groups. Due to the high lipophilic nature of PB, excretion and metabolism are most likely occur through the liver rather than the kidneys [70].

G-4 mice showed low glucose concentration in blood samples that could be due to the high presence of drugs in adipose tissues, which is the primary site for glucose metabolism [71]. In white adipose tissues, there is usually a high level of insulin sensitivity, which results in higher expression of glucose transporter protein such as glucose transporter type-4 (GLUT4), which increases glycaemic control [72]. The cause of significant variation in PB concentrations in skeletal muscle remains unclear and interesting, and further studies to investigate this variability will be conducted in the future. The excretion of high PB concentration in faeces from all groups, with significantly more form G-4, indicates that PB-LCA microcapsules could be helpful in the treatment of inflammatory bowel diseases through controlled release. The anti-inflammatory properties of PB have already been indicated as a possible treatment for inflammatory bowel diseases [69,73]. Fukami T et al. (2009) proved that nanotechnology enhances PB absorption and cellular permeation significantly [74]. Furthermore, Zhang Z et al. established that cellular uptake of PB could be improved by using nanoparticles with surfactants [75]. Thus, these results indicate that the higher PB concentration in blood and tissues is due to improved absorption and cellular permeation of PB when microencapsulated with bile acid, which correlates with reduced pro-inflammatory cytokines and improved blood glucose levels, survival rate, and diabetes signs and symptoms.

## 5. Conclusions

Our findings proved that the microencapsulation method we developed is robust and successful in producing microcapsules with uniform morphology, physiochemical compatibility, and membrane stability. The in vitro findings indicate significant positive biological effects of PB on pancreatic β-cells when LCA is incorporated into microcapsules. The study also examined PB-LCA microcapsules in vivo, which showed promising results in controlling signs and symptoms of diabetes, improving survival rate, hypoglycaemic effect, and increased drug absorption and bioavailability, which overall suggests potential application in T2D therapy.

## Figures and Tables

**Table 1:** Zeta potential, particle size analysis, and Fourier transform infrared. *N* = 3, mean ± SD.

Formulation	Zeta Potential (mV)	Particle Size (nm)	Fourier Transform Infrared Spectra (λ cm^−1^)
**F1**	63 ± 2	600 ± 10	3298, 1416, 1309, 1024
**F2**	67 ± 3	585 ± 5	3340, 1418, 1309.3, 1023

**Table 2:** Rheological parameter measurement before microencapsulation at various mixing speeds: *N* = 3, mean ± SD.

Formula Code	Speed	RPM	Torque (m Nm)
**F1**	1 2 3 4 5	35 107 187 572 1000	1.32 ± 0.3 1.42 ± 0.8 1.84 ± 0.4 2.78 ± 0.9 3.42 ± 0.28
**F2**	1 2 3 4 5	35 107 187 572 1000	1.24 ± 0.4 1.33 ± 0.22 1.88 ± 0.23 2.30 ± 0.4 2.82 ± 0.33

## Data Availability

The data presented in this study are available on request from the corresponding author.
